# Associations of Abnormal Sleep Duration and Chronotype with Higher Risk of Incident Amyotrophic Lateral Sclerosis: A UK Biobank Prospective Cohort Study

**DOI:** 10.3390/biomedicines13010049

**Published:** 2024-12-28

**Authors:** Gan Zhang, Wen Cao, Zhuoya Wang, Kailin Xia, Binbin Deng, Dongsheng Fan

**Affiliations:** 1 Department of Neurology, Peking University Third Hospital, Beijing 100191, China; g.zhang@bjmu.edu.cn (G.Z.); 2116393043@bjmu.edu.cn (W.C.); wangzhuoya@bjmu.edu.cn (Z.W.); kllook@pku.edu.cn (K.X.); 2 Beijing Municipal Key Laboratory of Biomarker and Translational Research in Neurodegenerative Diseases, Beijing 100191, China; 3 Department of Neurology, First Affiliated Hospital of Wenzhou Medical University, Wenzhou 325015, China; 4 Key Laboratory for Neuroscience, National Health Commission, Ministry of Education, Peking University, Beijing 100871, China

**Keywords:** sleep duration, chronotype, amyotrophic lateral sclerosis, UK Biobank

## Abstract

**Background:** The occurrence of sleep disturbances in amyotrophic lateral sclerosis (ALS) patients is widely reported. However, there is still a lack of reliable evidence of a relationship between sleep disturbances and the risk of developing ALS. The aim of this study was to prospectively investigate the longitudinal associations between sleep traits and the risk of incident ALS. **Methods:** We included information from 409,045 individuals from the prospective cohort of the UK Biobank. Sleep traits at baseline were measured using a standardized questionnaire. All sleep traits were analyzed in relation to the subsequent incidence of ALS using Cox proportional hazards models. **Results:** Multivariate analysis showed that 6–7 h of sleep was related to the lowest risk for ALS. A long sleep duration (≥8 h) was associated with an increased risk of ALS incidence (HR: 1.31, 95% CI: 1.07–1.61; *p* = 0.009). A short sleep duration (<6 h) was associated with an increased risk of ALS incidence (HR: 1.91, 95% CI: 1.10–3.30, *p* = 0.021) in females. In participants aged ≥65 years, eveningness was associated with increased ALS risk (HR: 1.32, 95% CI: 1.08–1.61; *p* = 0.006). **Conclusion:** Our results hint at a sleep duration that is too short or too long, and certain chronotypes might be related to the risk of developing ALS. Despite the limitations imposed by the study design and the subjectivity of sleep information, our findings suggest that sleep disturbances may influence the risk of developing ALS.

## 1. Introduction

Amyotrophic lateral sclerosis (ALS) is a fatal disease that affects motor neurons, leading to progressive limb weakness, dysphagia, and respiratory failure [1]. In the treatment of ALS, limited medications and supportive treatment can extend the survival time of patients to a certain extent [2,3,4,5]. However, none of these treatments can effectively reverse or terminate disease progression. For patients with ALS, it is still difficult to eliminate the possibility of respiratory failure and loss of systemic motor function, imposing a heavy burden on these patients, their caregivers, and even society. In this context, the exploration of risk factors for the development of ALS to guide prevention in at-risk populations, as well as the exploration of more potentially effective ALS intervention targets, is critical and in line with current clinical needs.

Sleep disturbances are indisputably common in patients with ALS, as early studies have reported that the proportion of ALS patients with sleep disturbances is much greater than that of the general population. Among them, abnormal sleep duration, difficulty falling asleep, low sleep efficiency, frequent awakening, and daytime sleepiness are the main sleep problems faced by ALS patients [6,7,8]. Especially in terms of sleep duration, previous reports have found that patients with ALS tend to have remarkably shorter sleep durations than the recommended sleep duration for adults [9,10]. It was previously believed that sleep disturbances are secondary changes caused by dyspnea, pain, and postural dysregulation caused by the motor symptoms of ALS [11]. However, increasing evidence of the adverse effects of sleep disturbances in neurodegenerative diseases has been presented [12,13]. Therefore, sleep disturbances may also contribute to the development of ALS.

Our previous study detected a causal relationship between genetically predicted sleep disturbances and a high risk of developing ALS [14]. However, population studies are needed to further verify the relationship we have found. Owing to the low incidence of ALS, no longitudinal studies have been conducted to further support the relationship between sleep disturbances and the risk of developing ALS, and whether sleep disturbances increase the incidence of ALS is not completely clear. Currently, the emergence of the UK Biobank, which is a large database that includes information from hundreds of thousands of individuals, enables the possibility of further exploring the associations between sleep traits and the risk of developing ALS. Accordingly, we utilized the dataset of a population-based prospective cohort from the UK Biobank and extracted data on sleep traits to explore whether there is a potential relationship between sleep traits and the incidence of ALS. Our study may provide useful information for ALS prevention and for identifying potential therapeutic targets for this disease.

## 2. Materials and Methods

### 2.1. Study Population

The data we included in our analysis were all from the UK Biobank, which is a prospective population-based cohort that includes information from approximately 500,000 adult individuals recruited between 2006 and 2010 in the United Kingdom to explore the genetic and lifestyle causes of diseases [15]. The UK Biobank was approved by the UK National Health Service, National Research Ethics Service North West, the National Information Governance Board for Health and Social Care in England and Wales, and the Community Health Index Advisory Group in Scotland. Data on the life habits, physical measurements, sociodemographic characteristics, and lifestyle exposures of all participants were collected from questionnaires at the recruitment visit. All participants provided written informed consent prior to inclusion in the study. For detailed information about the UK Biobank, see https://www.ukbiobank.ac.uk/, accessed on 16 August 2023. Our analysis included all enrolled UK Biobank patients, excluding those who had been diagnosed with ALS at baseline and those with incomplete sleep information.

### 2.2. Ascertainment of Sleep Traits

We extracted the questionnaire-sourced information on sleep duration, insomnia status, daytime napping status, difficulty getting up in the morning, snoring status, daytime sleepiness status, and chronotype obtained at the first visit. Each question had multiple-choice responses; a response of “Prefer not to answer” or “Do not know” was considered invalid, indicating missing data, and was excluded from subsequent analysis.

Sleep duration was determined with a self-reported answer to a standardized question: “About how many hours of sleep do you get every 24 h? (including naps)”, with the possible answers increasing in hourly integral values. Participants were asked to confirm their answer if a response was <3 or >12. Any responses of less than 1 h or greater than 23 h were considered unreliable and rejected. A total of 501,262 people answered the questions, of which 497,974 gave valid answers. The mean length of sleep was 7.152 ± 1.111 h.

Insomnia and daytime napping status were assessed with the following questions: “Do you have trouble falling asleep at night, or do you wake up in the middle of the night?” and “Do you nap during the day?” respectively. Each question has three ordinal response options: “Never/rarely”, “Sometimes”, “Usually”, and one invalid response, “Prefer not to answer”. A total of 501,258 people provided information about insomnia and daytime napping. Among them, 120,669 people reported never or rarely, 238,682 people sometimes, and 141,295 usually had insomnia. Additionally, 280,857 people never or rarely napped, 192,523 people napped sometimes, and 26,877 people usually napped.

Difficulty getting up in the morning was assessed by the following question: “On an average day, how easy do you find getting up in the morning?” with 4 ordinal answers: “Not at all easy”, “Not very easy”, “Fairly easy”, “Very easy”, and 2 invalid answers: “Do not know” and “Prefer not to answer”. A total of 497,495 people provided information on this question. Among them, 19,740 people reported that it was not at all easy, 69,878 people said it was not very easy, 246,473 people found it fairly easy, and 160,389 people found it very easy to get up.

Snoring status was assessed through the question “Does your partner or a close relative or friend complain about you snoring?” with 2 valid answers: “Yes” and “No”, and 2 invalid answers: “Do not know” and “Prefer not to answer”. A total of 501,258 participants responded. Of these, 173,269 people reported snoring, 291,857 did not snore, and 36,132 did not know if they snore.

Daytime sleepiness status was assessed through the question “How likely are you to doze off or fall asleep during the daytime when you do not mean to? (e.g., when working, reading or driving)” with 4 ordinal answers: “Never/rarely”, “Sometimes”, “Often”, “All of the time”, and 2 invalid answers: “Do not know” and “Prefer not to answer”. A total of 501,257 people answered this question. Of these, 378,428 people reported never or rarely, 105,899 sometimes, 14,045 often, and 2,057 people felt sleepy all the time.

Chronotype was assessed through the question “Do you consider yourself to be?” with one of the 4 ordinal answers: “Definitely a ‘morning’ person”, “More a ‘morning’ than an ‘evening’ person”, “More an ‘evening’ than a ‘morning’ person”, “Definitely an ‘evening’ person”, “Do not know”, or “Prefer not to answer”. A total of 497,493 people answered this question. Among them, 120,273 people considered themselves “Definitely a ‘morning’ person”, 157,285 people “More a ‘morning’ than an ‘evening’ person”, 126,255 people “More an ‘evening’ than a ‘morning’ person”, and 40,064 people “Definitely an ‘evening’ person”.

If their answers varied regarding sleep duration, insomnia, difficulty getting up, and chronotype, the participants were asked to give the average time they spent sleeping in the last four weeks. For snoring and daytime sleepiness, the participants were asked to provide an estimate or select “Do not know” if unsure.

### 2.3. Incident ALS

A follow-up for assessing the incidence of ALS was conducted from recruitment until September 16, 2021. The end of the follow-up period was defined as the time at which the patient was diagnosed with ALS, died, or reached the above-mentioned time point, whichever occurred first. ALS diagnosis was exacted from Hospital Episode Statistics Admitted Patient Care, Scottish Morbidity Record and Patient Episode Data for Wales, which includes patients’ in-hospital records in England, Scotland, and Wales. The diagnosis of ALS was classified according to the International Classification of Diseases—10th Revision codes (G12.2). The term ALS was used as an umbrella term for a group of motor neuron-related disorders.

### 2.4. Collection of Baseline Characteristics

Data on age, gender, smoking and drinking status, body mass index (BMI), education level, and the Townsend deprivation index (TDI) were collected via questionnaires.

### 2.5. Statistical Analyses

The baseline characteristics of participants with and without ALS are reported as medians and interquartile ranges for continuous variables and as numbers and percentages for categorical variables. Mann-Whitney U tests were used to compare continuous variables, while categorical variables were compared via χ^2^ tests.

We used restricted cubic splines to explore whether there was a nonlinear relationship between sleep duration and ALS risk. Then, time-to-event analyses of sleep duration and incident ALS were performed using log rank test and Cox proportional hazards models. We compared less than 6 h and more than 7 h of sleep with 6–7 h of sleep. The analyses of other sleep traits and incident ALS were also performed using Cox proportional hazards models. Insomnia status, daytime napping status, difficulty getting up in the morning, chronotype, and daytime sleepiness were included as ordinal variables in subsequent analyses. The presented hazard ratios (HRs) had a predictive effect on incident ALS per change in their level. Snoring was conducted for the subsequent analysis of the answers as binary variables. In the Cox proportional hazards models, Model 1 was adjusted for demographic variables, including age at recruitment and sex. Model 2 was further adjusted for education and the TDI, smoking status, drinking status, and BMI. The mathematical formulas are as follows: Model 1: $h\left(t|X\right)=h0\left(t\right)\;*\;exp(\beta 1\;*\;Sleep\;traits+\beta 2\;*\;Sex+\beta 3\;*\;Age)$; Model 2: $h\left(t|X\right)=h0\left(t\right)\;*\;exp(\beta 1\;*\;Sleep\;traits+\beta 2\;*\;Sex+\beta 3\;*\;Age+\beta 4\;*\;TDI+\beta 5\;*\;Education+\beta 6\;*\;BMI+\beta 7\;*\;Smoking+\beta 8\;*\;Alcohol)$.

We then performed a range of sensitivity analyses to test the robustness of our results. Neurodegenerative diseases are characterized by mild central nervous system lesions in patients before the onset of major symptoms. Therefore, abnormalities observed shortly before disease onset may reflect the pathological changes of the disease itself rather than independent exposure. This could confound the potential causality suggested by the analysis. Therefore, at first, participants with a short interval between baseline information collection and ALS diagnosis (1 year and 2 years) were excluded step by step with the purpose of excluding reverse causality as much as possible. Second, we excluded participants with extreme sleep durations (<3 and ≥13 h), which may reflect very short-term or inaccurate sleep durations.

Further analyses were performed between the subgroups separated by age (<65 years and ≥65 years), sex, and BMI at recruitment (<25 and ≥25). The interaction analysis of age, sex, and BMI subgroup was used to explore whether a stratum effect was present.

All of the above statistical analyses were performed using R (version 4.2.2; R Foundation for Statistical Computing, Vienna, Austria) and R packages (“survival” and “survminer” packages). The code we used in our analysis is shown in Table S1.

## 3. Results

### 3.1. Baseline Characteristics

**Figure 1:**
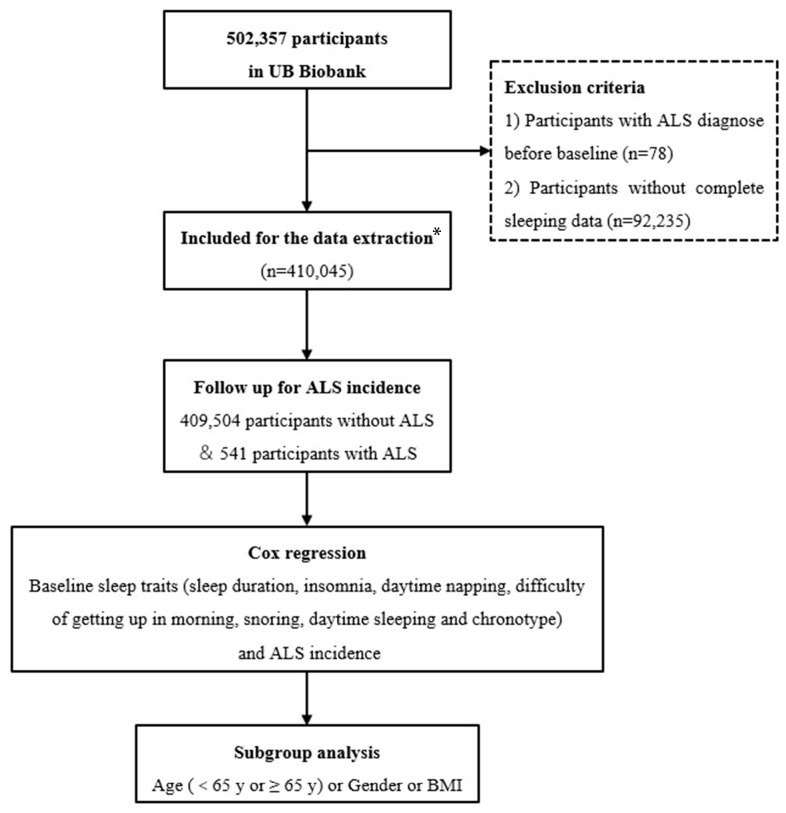
Flow chart of the process of the study. Participants with ALS at baseline and without complete sleep data were removed from the Cox proportional hazard analysis. * One participant with an ALS diagnosis before baseline without complete sleep data.

Data from 502,357 people from the UK Biobank were extracted. After 78 participants with ALS diagnosed before baseline and 92,235 participants without complete sleep data were excluded, a total of 410,045 eligible participants were included in this study. A total of 541 (0.13%) patients were diagnosed with ALS during the follow-up period. The study design is shown in Figure 1.

Compared with participants who were free from ALS during follow-up, the participants who were diagnosed with ALS were older and had a greater proportion of men and smokers (Table 1). The proportions of participants with sleep durations of <6 h and ≥8 h and a daytime napping habit were greater among the participants who were diagnosed with ALS during the follow-up period. Difficulty getting up in the morning also differed between the participants who were diagnosed with ALS during the follow-up period and those who were not (Table 2).

### 3.2. Associations Between Sleep Traits and the Incidence of ALS

**Figure 2:**
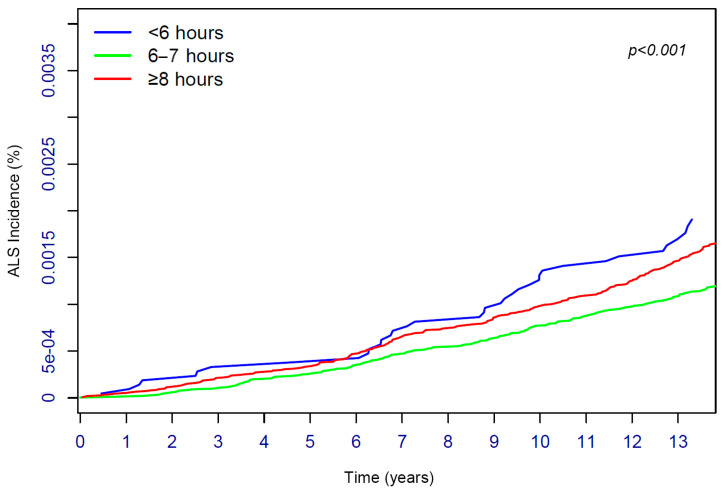
Kaplan–Meier curve of development of ALS during follow-up of participants with different sleep duration. The incidence of ALS in the 3 sleep duration groups was compared using log rank test. ALS = amyotrophic lateral sclerosis. The analyses were performed using log rank test.

We detected a U-shaped relationship between sleep duration and the incidence of ALS via restricted cubic spline analyses (*p* for nonlinearity: 0.006). In the log rank test, we found differences in the risk of ALS among sleep durations of <6 h, 6–7 h, and ≥8 h, and the 6–7 h group had the lowest risk (*p* < 0.001). Furthermore, we found that sleep durations of both <6 h and ≥8 h were associated with a greater risk of incident ALS in Model 1 of Cox proportional hazards analysis (sleep < 6 h: HR: 1.21, 95% CI: 1.02–1.44, *p* = 0.034; sleep ≥ 8 h: HR: 1.49, 95% CI: 1.06–2.10, *p* = 0.023). After further adjusting for other socioeconomic indicators in Model 2, a sleep duration of ≥8 h was also associated with an increased risk of developing ALS (HR: 1.31, 95% CI: 1.07–1.61, *p* = 0.009); however, the association between a sleep duration of < 6 h and the risk of developing ALS disappeared (HR: 1.44, 95% CI: 0.93–2.25, *p* = 0.104). No significant associations were observed between insomnia, daytime napping, difficulty getting up in the morning, snoring, daytime sleepiness, chronotype, and the incidence of ALS (*p* > 0.05). The restricted cubic spline results are shown in Figure S1. The development of ALS during follow-up for participants with different sleep durations (log rank test) is shown in Figure 2. The associations between sleep traits and ALS risk (Cox proportional hazards analysis) are shown in Table 3. Plots of Schoenfeld residuals for each covariate are shown in Figure S2.

### 3.3. Sensitivity Analyses

Participants with a short interval between baseline information collection and ALS diagnosis (1 year and 2 years) were excluded step by step with the aim of excluding reverse causality, which did not substantially affect the results (Table S2). Consistent direction and magnitude associations were also observed in the analysis of sleep duration after the exclusion of unreasonable values (Table S2).

### 3.4. Sleep Traits and ALS Risk Across Different Ages, Sexes, and BMIs

Considering that sleep is affected by a variety of factors, we performed a series of subgroup analyses to further explore whether other factors interact with the relationships between sleep traits and the incidence of ALS.

In participants younger than 65 years, a sleep duration of ≥8 h was associated with a greater risk of ALS incidence (HR: 1.49, 95% CI: 1.17–1.90; *p* = 0.001; Model 2). In addition, a sleep duration of <6 h was also associated with a greater risk of developing ALS; however, the correlation was not statistically significant (HR: 1.65, 95% CI: 0.99–2.73; *p* = 0.054). In females, <6 h of sleep was associated with a greater risk of ALS incidence (HR: 1.91, 95% CI: 1.10–3.30, *p* = 0.021), whereas this association was not present in males (HR: 0.96, 95% CI: 0.45–2.07, *p* = 0.914). In participants with a BMI ≥ 25, ≥8 h of sleep was associated with a greater risk of developing ALS (HR: 1.38, 95% CI: 1.07–1.78, *p* = 0.012); this association was also not present in participants with a BMI < 25 (HR: 1.19, 95% CI: 0.84–1.69, *p* = 0.324). In male participants older than 65 years and with BMI < 25, we did not find a correlation between sleep duration and incident ALS.

An analysis of other sleep traits revealed that in participants older than 65 years, eveningness was associated with increased ALS risk (HR: 1.32, 95% CI: 1.08–1.61; *p* = 0.006). However, we did not find such an association in participants younger than 65 years and sex or BMI subgroups.

In the sex, age, and BMI subgroups, we still found no associations between other sleep traits (insomnia status, daytime napping status, difficulty getting up in the morning, snoring status, and daytime sleepiness) and the incidence of ALS. The above results are shown in Table 4. Plots of Schoenfeld residuals for each covariate in the subgroup analysis are shown in Figures S3–S8.

## 4. Discussion

We analyzed the associations between multiple sleep traits and ALS risk. To our knowledge, our study is the most comprehensive and largest longitudinal study to investigate the associations between sleep traits and ALS risk. Our analyses of data from a large prospective UK Biobank cohort revealed the following key findings: (1) Sleep duration has a U-shaped relationship with ALS risk; that is, sleep durations that are too long or too short were associated with a greater risk of developing ALS, and BMI, age, and sex interacted with this relationship. (2) Chronotype was also associated with ALS risk, with people over 65 years of age with a nocturnal chronotype having a greater risk of developing ALS.

Sleep duration is the most intuitive index for evaluating patients’ sleep conditions. Current evidence suggests that a short sleep duration might increase the incidence of some neurodegenerative diseases, but no studies have demonstrated such a relationship between sleep traits and the risk of developing ALS, which is also a neurodegenerative disease, at the clinical level. According to recent studies, about 7 h of sleep has been confirmed as the best sleep duration for adults [16,17], and further studies have found that 6–7 h is the ideal sleep duration associated with the lowest risk of death from various diseases [18,19]. Similarly, in the restricted cubic splines, we found that the risk of developing ALS was lowest when the duration of sleep was 6–7 h, and the risk gradually increased with decreasing or increasing sleep duration. Therefore, in the follow-up analysis, we used 6–7 h as the reference value for sleep duration to compare the difference in the risk of developing ALS during follow-up between participants with ≥8 h of sleep and those with <6 h of sleep.

Although the association between <6-h sleep durations and the risk of developing ALS at baseline was not significant overall, after controlling for confounders, we found that there was a significant correlation in women. In recent years, mechanistic research has provided further evidence that short sleep durations increase the risk of developing ALS. The glymphatic system, a structure critical for clearing a variety of intracranial pathological deposits in neurodegenerative diseases, might be the link between short sleep duration and the risk of developing ALS [20,21,22]. The glymphatic system operates efficiently during sleep, suggesting that short sleep increases pathological intracranial deposition, which in turn accelerates the progression of neurodegenerative diseases [23]. ALS is also a neurodegenerative disease characterized by abnormal deposition of brain tissue, so a short sleep duration may also contribute to the onset and progression of ALS by affecting this structure [24,25]. In addition, short sleep durations are also associated with high levels of inflammation [26], which overlaps with the pathological changes observed in individuals with ALS [27]. Therefore, our results support the hypothesis that short sleep durations increase the risk of developing ALS. We observed different results across sex subgroups, suggesting that the effect of sleep duration on ALS risk is associated with sex.

Moreover, surprisingly, our study revealed a stronger association between ≥8 h of sleep at baseline and higher follow-up ALS incidence. This finding was stable after excluding participants who developed short latency between baseline and diagnosis, which further reduces the possibility of reverse causality. These results suggest that long sleep durations or related factors may increase the risk of developing ALS. There are several plausible explanations for the association between long sleep duration and the risk of developing ALS. First, the inflammatory response may be the biological mechanism underlying the observed associations. Several studies have shown that the progression of ALS is accompanied by an inflammatory response, particularly increased peripheral concentrations of IL-6 [28,29]. In addition, Irwin M.R. et al. reported that a long sleep duration was associated with increased levels of IL-6 [26]. These findings suggest that the inflammatory response might be the reason why long sleep durations increase the risk of these diseases. In addition, long sleep duration is often associated with inefficient sleep; that is, patients need longer sleep to compensate for the lack of physical recovery caused by low sleep efficiency. Therefore, our results suggest that low sleep efficiency may adversely affect ALS risk; however, we do not currently have data on low sleep efficiency to further test this possibility. We found that a long sleep duration was not associated with a greater risk of developing ALS in people older than 65 years and with a BMI less than 25, which may be due to the stratified effects of age and BMI.

However, there is another possibility to consider. Previous studies based on imaging or pathology have revealed that the pathology of ALS involves not only changes in the motor system but also changes in multiple areas in the central nervous system, including the hypothalamus and thalamus, which are sleep-related regions [24,25,30,31]. Therefore, abnormal sleep duration may be a manifestation of the broader and more severe pathology of ALS. The pathological changes in ALS occur long before motor symptoms are observed [32], and our findings suggest that short and long sleep durations may be early and sensitive manifestations of pathological changes in ALS.

In terms of other sleep characteristics, we also included insomnia in our analysis. The international classification of sleep disorders’ definition of insomnia is not restricted to assessments of the quality or quantity of sleep. The specific content involves a decline in sleep quality; sleep difficulty; sleep maintenance difficulties and other night-sleep changes; and sleepiness, inattention, and other secondary changes during the day, which can reflect a person’s comprehensive sleep situation and cover multiple sleep factors previously found to be associated with an increased risk of developing neurodegenerative diseases [33]. A recent study by Silva F et al. suggests that better sleep quality was associated with longer survival in ALS patients [34]. This finding suggests that insomnia may also increase the incidence of ALS. However, our study did not reveal a clear association between insomnia and the risk of developing ALS, and the simplicity of the problem of gathering information about insomnia may be the reason why the results do not match expectations. Studies of insomnia require more comprehensive information to judge the sleep status of participants. In addition, specific sleep disturbances (such as non-rapid eye movement sleep stage 3–4 or rapid eye movement sleep reduction) rather than overall sleep status may be associated with ALS risk, so follow-up studies may need to be further clarified with data from polysomnographic monitoring. Current evidence suggests that napping might have protective or harmful effects on the development of neurodegenerative diseases [35,36]. However, in these studies, it is unclear whether napping compensates for a lack of sleep at night or supplements a proper night’s sleep. Further studies on napping should be conducted while controlling for nighttime sleep conditions. In this study, we did not find an association between napping status and the risk of developing ALS. Our previous MR study revealed that daytime sleepiness is associated with an increased risk of developing ALS [14]; however, our current study did not reveal an association between daytime sleepiness and ALS risk, and whether there is an association between these variables at the clinical level remains uncertain.

The human circadian system is regulated by the suprachiasmatic nucleus located in the anterior hypothalamus. Previous studies have revealed that pathological changes in the hypothalamus of the ALS model involve key circadian regulators, suggesting that differences in circadian rhythm may also be manifestations of ALS-related pathological changes [37]. Our study revealed that a nocturnal chronotype was associated with a greater risk of developing ALS in people older than 65 years, supporting the above findings. We did not observe a similar association in participants younger than 65 years of age, which may be because these participants still needed to work, artificially influenced by their lifestyle mask actual changes in circadian rhythms [38], or circadian rhythm changes were only associated with ALS onset in old age.

Finally, previous studies have shown that patients with nocturnal apnea have a faster rate of disease progression, suggesting that the hypoxic state during sleep may have an adverse effect on ALS [34,39,40]. However, our study also did not reveal a correlation between nighttime snoring status, a breathing-related factor, and ALS risk, which may also be influenced by the oversimplification of the UK Biobank question used to assess snoring status. Most participants who snore may not have experienced nighttime hypoxia.

Our study has several strengths. First, owing to the geographical incidence of ALS, it is difficult to conduct cohort studies related to the incidence of ALS in the population. We carried out the relevant analysis using the data provided by the UK Biobank. To our knowledge, our study is the first large-sample prospective cohort study to examine the relationship between sleep traits and the incidence of ALS. Second, sleep is a complex state, and our study included multiple sleep-related indicators, which provide more comprehensive evidence for understanding the relationship between the risk of developing ALS and sleep traits.

However, our study has several limitations: (1) Despite being a prospective cohort, causation should be interpreted with caution, and our results also suggest that the presence of poor sleep is merely a manifestation of the pathological changes that precede the onset of ALS motor symptoms. (2) Although the results based on questionnaires cover all aspects of sleep, they cannot accurately represent the sleep conditions of patients. Future studies need more detailed and objective sleep data to explore this issue further. (3) We do not have long-term follow-up results for sleep-related data, and baseline data may only represent short-term sleep conditions that occurred at enrollment. It is not clear whether this status persisted or changed over time, which challenges the reliability of our results. (4) Due to the limitations of the original data, we were unable to correct for factors that may affect sleep state, such as hypnotics, as covariables. When conditions permit, we aim to further exclude the potential impact of these circumstances on our results. (5) While there are several mechanisms by which sleep disturbances may be associated with ALS risk, our study is only an observation of clinical phenomena and does not provide further evidence of the mechanisms. We propose that future studies exploring biological mechanisms would be valuable.

## 5. Conclusions

A sleep duration of 6–7 h was associated with the lowest ALS incidence, and a regular sleep duration that is too long or too short may lead to an increased risk of developing ALS. Although ALS is a rare disease with a very low incidence, people at high risk of the disease still need to be vigilant and receive timely intervention for these conditions. Monitoring sleep disturbances would have potential benefits for this population. Sleep duration and chronotype may also reflect the pathological changes associated with ALS. Future research directions include exploring the biological link between sleep traits and the risk of developing ALS more deeply and in greater detail, as well as searching for potential treatments to improve the quality of life and prognosis of people with ALS. Research in this area promises to further our understanding of the causes and progression of ALS, advance our understanding of the disease, and open new avenues for future treatment strategies.

## Figures and Tables

**Table 1:** Comparison of baseline variables between participants without incident ALS and those with incident ALS.

Variable	Without Incident ALS (*n* = 409,504)	With Incident ALS (*n* = 541)	*p*
Sex			<0.001 ***
Male	184,048 (44.9%)	296 (54.7%)	
Female	225,456 (55.1%)	245 (45.3%)	
Age (years)	58.0 [50.0, 63.0]	62.0 [58.0, 66.0]	<0.001 ***
BMI (kg/m^2^)	26.7 [24.1, 30.0]	26.7 [24.1, 30.0]	0.818
Smoking status			0.003 **
Never	222,593 (54.4%)	257 (47.5%)	
Previous	143,212 (35.0%)	226 (41.8%)	
Current	42,499 (10.4%)	57 (10.5%)	
Alcohol consumption status			0.642
Never	17,067 (4.2%)	21 (3.9%)	
Previous	14,125 (3.4%)	15 (2.8%)	
Current	378,031 (92.3%)	505 (93.3%)	
Education level			0.147
College or university degree	134,036 (32.7%)	153 (28.3%)	
A levels/AS levels or equivalent	45,965 (11.2%)	40 (7.4%)	
O levels/GCSEs or equivalent	87,497 (21.4%)	122 (22.6%)	
CSEs or equivalent	22,149 (5.4%)	29 (5.4%)	
NVQ, HND, HNC or equivalent	27,042 (6.6%)	30 (5.5%)	
Other professional qualifications	21,386 (5.2%)	29 (5.4%)	
Townsend deprivation index	−2.23 [−6.26, 11.0]	−2.21 [−6.00, 9.00]	0.817

Data are presented as numbers (%) for categorical variables and medians (Q1, Q3) for continuous variables. Abbreviations: ALS = amyotrophic lateral sclerosis; BMI = body mass index; age, BMI, smoking status, alcohol consumption status, education level, and Townsend deprivation index between the 2 groups were compared using the Mann-Whitney U test; sex between the 2 groups was compared using χ^2^ tests. ** *p* < 0.01; *** *p* < 0.001.

**Table 2:** Comparison of baseline sleep traits between participants without incident ALS and those with incident ALS.

**Variable**	**Without Incident ALS** **(*n* = 409,504)**	**With Incident ALS** **(*n* = 541)**	***p***
Sleep duration			0.001 **
<6 h	21,326 (5.2%)	37(23.7%)	
6–7 h	236,660 (57.8%)	272(33.5%)	
≥ 8 h	151,518 (37.0%)	232(42.9%)	
Insomnia status			0.699
Never/rarely	99,827 (24.4%)	127 (23.5%)	
Sometimes	195,791 (47.8%)	255 (47.1%)	
Usually	113,886 (27.8%)	159 (29.4%)	
Daytime napping status			0.029 *
Never/rarely	231,210 (56.5%)	280 (51.8%)	
Sometimes	156,735 (38.3%)	222 (41.0%)	
Usually	21,559 (5.3%)	39 (7.2%)	
Difficulty getting up in morning			0.041 *
Not at all easy	15,667 (3.8%)	22 (4.1%)	
Not very easy	57,597 (14.1%)	57 (10.5%)	
Fairly easy	203,032 (49.6%)	262 (48.4%)	
Very easy	133,208 (32.5%)	200 (37.0%)	
Snoring status			0.821
Yes	152,178 (37.2%)	198 (36.6%)	
No	257,326 (62.8%)	343 (63.4%)	
Daytime sleepiness status			0.930
Never/rarely	311,985 (76.2%)	407 (75.2%)	
Sometimes	86,307 (21.1%)	120 (22.2%)	
Often	11,189 (2.7%)	14 (2.6%)	
All of the time	23 (0.0%)	0 (0.0%)	
Chronotype			0.355
Definitely a “morning” person	110,895 (27.1%)	146 (27.0%)	
More a “morning” than “evening” person	146,054 (35.7%)	177 (32.7%)	
More a “evening” than “morning” person	116,435 (28.4%)	171 (31.6%)	
Definitely a “evening” person	36,120 (8.8%)	47 (8.7%)	

Data are presented as numbers (%) for categorical variables and medians (Q1, Q3) for continuous variables. Abbreviation: ALS = amyotrophic lateral sclerosis. All the sleep traits between the 2 groups were compared using the Mann-Whitney U test. * *p* < 0.05; ** *p* < 0.01.

**Table 3:** Sleep traits and ALS incidence.

Sleep Pattern	Model 1		Model 2	
	HR (95% CI)	*p*	HR (95% CI)	*p*
Sleep duration				
<6 h	1.49 (1.06–2.10)	0.023 *	1.44 (0.93–2.25)	0.104
6–7 h	Reference		Reference	
≥ 8 h	1.21 (1.02–1.44)	0.034 *	1.31 (1.07–1.61)	0.009 **
Insomnia status	1.02 (0.91–1.15)	0.736	1.01 (0.88–1.16)	0.913
Daytime napping status	1.00 (0.87–1.15)	0.999	0.97 (0.82–1.14)	0.683
Difficulty getting up in the morning	0.94 (0.84–1.06)	0.309	0.95 (0.83–1.09)	0.454
Snoring status	1.12 (0.94–1.34)	0.198	1.14 (0.92–1.41)	0.220
Daytime sleepiness status	0.91 (0.76–1.07)	0.251	0.91 (0.74–1.12)	0.385
Chronotype	1.09 (1.00–1.20)	0.057	1.08 (0.97–1.20)	0.166

Abbreviations: ALS = amyotrophic lateral sclerosis; HR = hazard ratio; CI = confidence interval. * *p* < 0.05; ** *p* < 0.01. The analyses of sleep traits and incident ALS were performed using Cox proportional hazards models.

**Table 4:** Sleep traits and ALS incidence stratified by age, sex, and BMI.

Sleep Trait	Age		Sex		BMI	
	<65 years (*n* = 332,717; *n* of ALS = 354) HR (95% CI), *p*	≥65 years (*n* = 77,328; *n* of ALS = 187) HR (95% CI), *p*	Male (*n* = 184,344; *n* of ALS = 296) HR (95% CI), *p*	Female (*n* = 225.701; *n* of ALS = 245) HR (95% CI), *p*	<25 (*n* = 135,542; *n* of ALS = 174) HR (95% CI), *p*	≥25 (*n* = 272,528; *n* of ALS = 359) HR (95% CI), *p*
Sleep duration						
<6 h	1.65 (0.99–2.73), 0.054	1.02 (0.41–2.55), 0.969	0.96 (0.45–2.07), 0.914	**1.91 (1.10**–3.30), 0.021 *	1.55 (0.71–3.37), 0.273	1.40 (0.82–2.40), 0.219
6–7 h	Reference	Reference	Reference	Reference	Reference	Reference
≥ 8 h	**1.49 (1.17**–1.90), 0.001 **	0.96 (0.66–1.40), 0.839	1.30 (0.98–1.73), 0.073	1.34 (1.00–1.79), 0.053	1.19 (0.84–1.69), 0.324	**1.38 (1.07**–1.78), 0.012 *
Insomnia status	0.98 (0.84–1.13), 0.741	1.11 (0.91–1.36), 0.318	1.03 (0.88–1.21), 0.679	1.01 (0.84–1.21), 0.954	1.00(0.81–1.23), 0.964	1.03 (0.89–1.20), 0.658
Daytime napping status	0.99 (0.81–1.22), 0.935	0.93 (0.69–1.25), 0.633	0.91 (0.73–1.14), 0.415	1.05 (0.81–1.35), 0.725	0.92 (0.68–1.24), 0.563	0.99 (0.81–1.21), 0.901
Difficulty getting up in the morning	0.95 (0.82–1.11), 0.538	0.94 (0.72–1.21), 0.619	0.93 (0.77–1.13), 0.447	0.97 (0.81–1.17), 0.758	0.92 (0.74–1.16), 0.499	0.97 (0.82–1.14), 0.670
Snoring status	1.09 (0.87–1.37), 0.447	1.17 (0.85–1.60), 0.333	1.10 (0.87–1.40), 0.418	1.10 (0.82–1.47), 0.523	0.98 (0.69–1.37), 0.889	1.16 (0.93–1.43), 0.189
Daytime sleepiness status	0.93 (0.75–1.16), 0.534	0.88 (0.67–1.16), 0.367	0.81 (0.64–1.02), 0.074	1.05 (0.82–1.35), 0.699	0.79 (0.57–1.12), 0.184	0.95 (0.78–1.16), 0.610
Chronotype	1.00 (0.88–1.13), 0.968	**1.32 (1.08**–1.61), 0.006 **	1.15 (0.99–1.33), 0.074	1.01 (0.87–1.18), 0.886	1.18 (0.98–1.42), 0.082	1.04 (0.91–1.18), 0.597

Abbreviations: ALS = amyotrophic lateral sclerosis; BMI = body mass index; HR = hazard ratio; CI = confidence interval. * *p* < 0.05; ** *p* < 0.01. The analyses of sleep traits and incident ALS were performed using Cox proportional hazards models.

## Data Availability

The data supporting the findings of this study are available on the UK Biobank project site and are subject to successful registration and application processes. Further details are available at https://www.ukbiobank.ac.uk/.

## Supplementary Materials

The following supporting information can be downloaded at https://www.mdpi.com/article/10.3390/biomedicines13010049/s1. Table S1. The R code used in analysis; Table S2. Sensitivity analysis of the effects of sleep traits on the incidence of ALS; Figure S1: Nonlinear relationship between sleep duration and the risk of developing ALS (restricted cubic splines); Figure S2: Schoenfeld residual plot of covariates in the Cox proportional hazards analysis (Model 2); Figure S3: Schoenfeld residual plot of covariates in Cox proportional hazards analysis for participants younger than 65 years (Model 2); Figure S4: Schoenfeld residual plot of covariates in Cox proportional hazards analysis for participants older than 65 years (Model 2); Figure S5: Schoenfeld residual plot of covariates in Cox proportional hazards analysis in men (Model 2); Figure S6: Schoenfeld residual plot of covariates in Cox proportional hazards analysis for women (Model 2); Figure S7: Schoenfeld residual plot of covariates in Cox proportional hazards analysis for participants with BMI less than 25 (Model 2); Figure S8: Schoenfeld residual plot of covariates in Cox proportional hazards analysis in participants with BMI greater than 25 (Model 2).
