# Supplementary material for: Associations of Abnormal Sleep Duration and Chronotype with Higher Risk of Incident Amyotrophic Lateral Sclerosis: A UK Biobank Prospective Cohort Study
Source: Biomedicines. 2024 Dec 28;13(1):49. doi: 10.3390/biomedicines13010049 (PMC11762514; doi:10.3390/biomedicines13010049)
Supplement: Supplementary file 1 [file biomedicines-13-00049-s001.zip › Table S1.pdf]

Table S1. The R code used in analysis.

```
setwd("D:/UKB-SLEEP")
a<-read.csv("D:/UKB-SLEEP/a3(2).csv",header = T)
names(a)
a$Sleep.duration <- as.numeric(a$Sleep.duration)
cox<-coxph(Surv(Latency,ALS)~Sleep.duration,data = a);summary(cox);AIC(cox)

a$sdc<-cut(a$Sleep.duration,
           breaks = c(-Inf, 6, 9, Inf),
           labels = c("1", "2", "3"),
           right = FALSE)
a$sdc<-relevel(a$sdc,ref="2")
library(survival)
cox<-coxph(Surv(Latency,ALS)~sdc+Sex+Age,data = a);summary(cox);AIC(cox)
cox<-coxph(Surv(Latency,ALS)~sdc+Sex+Age+TDI+Qualifications,data =
a);summary(cox);AIC(cox)
cox<-
coxph(Surv(Latency,ALS)~sdc+Sex+Age+TDI+Qualifications+BMI+Smoking+Alcohol,data
= a);summary(cox);AIC(cox)
cox<-coxph(Surv(Latency,ALS)~Sleeplessness.insomnia+Sex+Age,data =
a);summary(cox);AIC(cox)
cox<-coxph(Surv(Latency,ALS)~Nap.during.day+Sex+Age,data =
a);summary(cox);AIC(cox)
cox<-coxph(Surv(Latency,ALS)~Getting.up.in.morning+Sex+Age,data =
a);summary(cox);AIC(cox)
cox<-coxph(Surv(Latency,ALS)~Snoring+Sex+Age,data = a);summary(cox);AIC(cox)
cox<-coxph(Surv(Latency,ALS)~Daytime.dozing+Sex+Age,data =
a);summary(cox);AIC(cox)
cox<-coxph(Surv(Latency,ALS)~Morning.evening.person.chronotype+Sex+Age,data =
a);summary(cox);AIC(cox)
cox<-
coxph(Surv(Latency,ALS)~Sleeplessness.insomnia+Sex+Age+TDI+Qualifications+BMI+Sm
oking+Alcohol,data = a);summary(cox);AIC(cox)
cox<-
coxph(Surv(Latency,ALS)~Nap.during.day+Sex+Age+TDI+Qualifications+BMI+Smoking+
Alcohol,data = a);summary(cox);AIC(cox)
cox<-
coxph(Surv(Latency,ALS)~Getting.up.in.morning+Sex+Age+TDI+Qualifications+BMI+Sm
oking+Alcohol,data = a);summary(cox);AIC(cox)
cox<-
coxph(Surv(Latency,ALS)~Snoring+Sex+Age+TDI+Qualifications+BMI+Smoking+Alcohol,
data = a);summary(cox);AIC(cox)
cox<-
coxph(Surv(Latency,ALS)~Daytime.dozing+Sex+Age+TDI+Qualifications+BMI+Smoking+
```

```
Alcohol,data = a);summary(cox);AIC(cox)
cox<-
coxph(Surv(Latency,ALS)~Morning.evening.person.chronotype+Sex+Age+TDI+Qualifications+BMI+Smoking+Alcohol,data = a);summary(cox);AIC(cox)
```

```
# Plots of Schoenfeld residuals ---------------------------------------------
library(survival)
cox.zph_result <- cox.zph(cox)
print(cox.zph_result)
plot(cox.zph_result)
pdf("Schoenfeld_residuals.pdf")
plot(cox.zph_result)
dev.off()
png("Schoenfeld_residuals.png", width = 800, height = 600)
plot(cox.zph_result)
dev.off()
```

```
# RCS curve-------------------------------------------------------------
install.packages("rms")
library(rms)
dd <- datadist(a)   # 'a'
options(datadist = "dd")

cox_rcs <- cph(Surv(Latency, ALS) ~ rcs(Sleep.duration, 4) + Sex + Age + TDI + Qualifications + BMI + Smoking + Alcohol,
                 data = a,
                 x = TRUE, y = TRUE)
anova(cox_rcs)
```

```
# subgroup -------------------------------------------------------------
#sex
cox<-coxph(formula = Surv(Latency,ALS)~sdc+Sex+Age+TDI+Qualifications+BMI+Smoking+Alcohol,data = a[a$Sex ==0,]);summary(cox);AIC(cox)
cox<-coxph(formula = Surv(Latency,ALS)~sdc+Sex+Age+TDI+Qualifications+BMI+Smoking+Alcohol,data = a[a$Sex ==1,]);summary(cox);AIC(cox)
  #age
a$sage<-ifelse(a$Age<65,"1","2")
cox<-coxph(formula = Surv(Latency,ALS)~sdc+Sex+Age+TDI+Qualifications+BMI+Smoking+Alcohol,data = a[a$sage ==1,]);summary(cox);AIC(cox)
cox<-coxph(formula = Surv(Latency,ALS)~sdc+Sex+Age+TDI+Qualifications+BMI+Smoking+Alcohol,data = a[a$sage ==2,]);summary(cox);AIC(cox)
#BMI
a$SBMI<-ifelse(a$BMI<25,"1","2")
```

```
cox<-coxph(formula =
Surv(Latency,ALS)~sdc+Sex+Age+TDI+Qualifications+BMI+Smoking+Alcohol,data =
a[a$SBMI ==1,]);summary(cox);AIC(cox)
cox<-coxph(formula =
Surv(Latency,ALS)~sdc+Sex+Age+TDI+Qualifications+BMI+Smoking+Alcohol,data =
a[a$SBMI ==2,]);summary(cox);AIC(cox)
names(a)
```

```
# sensitivity analyses -----------------------------------------------------
a2 <- subset(a, a$ALS == "1" & a$Latency > 365)
b <- subset(a, a$ALS == 0)
a2<-rbind(a2,b)
a2$sdc<-relevel(a2$sdc,ref="2")
cox<-
coxph(Surv(Latency,ALS)~Daytime.dozing+Sex+Age+TDI+Qualifications+BMI+Smoking+
Alcohol,data = a2);summary(cox);AIC(cox)

a2 <- subset(a, a$ALS == "1" & a$Latency > 730)
b <- subset(a, a$ALS == 0)
a2<-rbind(a2,b)
a2$sdc<-relevel(a2$sdc,ref="2")
cox<-
coxph(Surv(Latency,ALS)~Daytime.dozing+Sex+Age+TDI+Qualifications+BMI+Smoking+
Alcohol,data = a2);summary(cox);AIC(cox)

a3 <- subset(a, a$ALS == "1" & a$Latency > 1095)
b <- subset(a, a$ALS == 0)
a3<-rbind(a3,b)
a3$sdc<-relevel(a3$sdc,ref="2")
cox<-
coxph(Surv(Latency,ALS)~Daytime.dozing+Sex+Age+TDI+Qualifications+BMI+Smoking+
Alcohol,data = a3);summary(cox);AIC(cox)

install.packages("mice")
library(mice)
str(a)
a[] <- lapply(a, function(x) if(is.factor(x) || is.character(x)) as.numeric(as.character(x)) else x)

a_standardized <- scale(a)
a4 <- mice(a_standardized,m=5,method=c("pmm")) ， m=5
a4 <- complete(a4)
a4$sdc<-relevel(a4$sdc,ref="2")
cox<-
coxph(Surv(Latency,ALS)~sdc+Sex+Age+TDI+Qualifications+BMI+Smoking+Alcohol,data
= a4);summary(cox);AIC(cox)
```

```
library(dplyr)

filtered_data <- a %>% filter(SBMI == 2)

als_summary <- filtered_data %>%
  group_by(ALS) %>%
  summarise(count = n())

print(als_summary)
```

```
# baseline ------------------------------------------------------------
library(boot)
library(table1)
a$Sex <- factor(a$Sex)
a$ALS <- factor(a$ALS)
a$Smoking <- factor(a$Smoking)
a$Alcohol <- factor(a$Alcohol)
a$Getting.up.in.morning <- factor(a$Getting.up.in.morning)
a$Nap.during.day <- factor(a$Nap.during.day)
a$Sleeplessness.insomnia <- factor(a$Sleeplessness.insomnia)
a$Snoring <- factor(a$Snoring)
a$Daytime.dozing <- factor(a$Daytime.dozing)
a$Qualifications <- factor(a$Qualifications)
a$sdc <- factor(a$sdc)
table1(~Sex+Age+BMI+TDI+Qualifications+Smoking+Alcohol+Sleep.duration+sdc+Getting.up.in.morning+Nap.during.day+Sleeplessness.insomnia+Snoring+Daytime.dozing|ALS,data = a,extra.col=list(`P-value`=pvalue),overall = F,render.continuous=render.median)
table(a$sdc)

able <- table(a$ALS, a$sdc)
chisq.test(table)

anova_result <- aov(sdc ~ ALS, data = a)
summary(anova_result)
tukey_result <- TukeyHSD(anova_result)
print("Tukey HSD Test Results:")
print(tukey_result)

table(a$Sleep.duration)

a4 <- subset(a, Sleep.duration > 2 & Sleep.duration < 13)
a4$sdc<-relevel(a4$sdc,ref="2")
cox<-
coxph(Surv(Latency,ALS)~sdc+Sex+Age+TDI+Qualifications+BMI+Smoking+Alcohol,data = a4);summary(cox);AIC(cox)
```

Qualifications: education level.
