# Supplementary material for: Associations of Abnormal Sleep Duration and Chronotype with Higher Risk of Incident Amyotrophic Lateral Sclerosis: A UK Biobank Prospective Cohort Study
Source: Biomedicines. 2024 Dec 28;13(1):49. doi: 10.3390/biomedicines13010049 (PMC11762514; doi:10.3390/biomedicines13010049)
Supplement: Supplementary file 1 [file biomedicines-13-00049-s001.zip › Table S2.pdf]

Supplementary Table S2. Sensitivity analysis of the effects of sleep traits on the incidence of ALS.

Sleep traits	Sensitive analysis 1 (n=335,742; n of ALS=388)		Sensitive analysis 2 (n=335,727; n of ALS=373)		Sensitive analysis 3 (n=335,555; n of ALS=396)	
	HR (95% CI)	p	HR (95% CI)	p	HR (95% CI)	p
Sleep duration						
< 6 hours	1.38(0.88,2.17)	0.162	1.30(0.81,2.08)	0.284	1.45(0.93,2.26)	0.097
6-7 hours						
≥ 8 hours	1.26(1.03,1.56)	0.026*	1.26(1.02,1.56)	0.030*	1.31(1.06,1.60)	0.011*
Insomnia status	0.99(0.86,1.14)	0.921	0.95(0.83,1.10)	0.513	/	/
Daytime napping status	0.96(0.81,1.14)	0.674	0.97(0.82,1.15)	0.725	/	/
Difficulty getting up in the morning	0.99(0.84,1.10)	0.521	0.95(0.83,1.09)	0.446	/	/
Snoring status	1.14(0.92,1.41)	0.248	1.13(0.91,1.41)	0.279	/	/
Daytime sleepiness status	0.90(0.73, 1.11)	0.348	0.90(0.73,1.11)	0.344	/	/
Chronotype	1.08(0.97, 1.21)	0.141	1.10(0.99,1.23)	0.084	/	/

Sensitive analysis 1 was an analysis of the effects of sleep traits on the incidence of ALS, with the exclusion of ALS patients whose latency was shorter than 1 year from initial information collection to diagnosis (1 year).

Sensitive analysis 2 was an analysis of the effects of sleep traits on the incidence of ALS, excluding ALS patients whose latency was shorter than 2 years from initial information collection to diagnosis (2 years).

Sensitive analysis 3 was an analysis of the effect of sleep duration on the incidence of ALS, excluding extreme values (<3 and ≥13 hours of sleep).

All sensitivity analyses were performed via the fully adjusted model (Model 2), which was adjusted for age, sex, BMI, education level, the Townsend deprivation index, smoking status and alcohol consumption status.

Abbreviations: ALS = amyotrophic lateral sclerosis; BMI = body mass index; HR = hazard ratio; CI = confidence interval; *p<0.05.
