# Supplementary figures and images for: Associations of Abnormal Sleep Duration and Chronotype with Higher Risk of Incident Amyotrophic Lateral Sclerosis: A UK Biobank Prospective Cohort Study
Source: Biomedicines. 2024 Dec 28;13(1):49. doi: 10.3390/biomedicines13010049 (PMC11762514; doi:10.3390/biomedicines13010049)

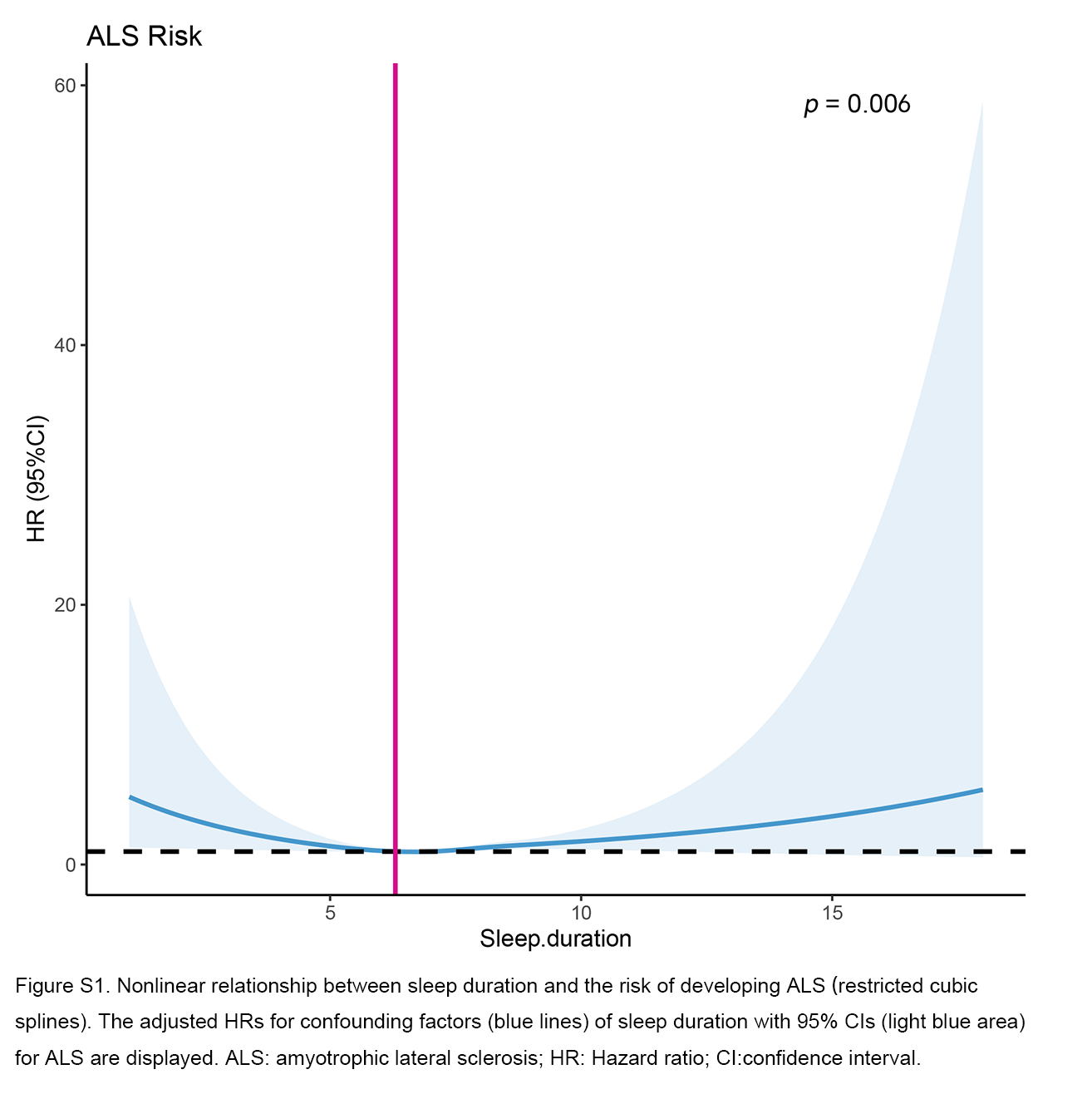

Supplement: Supplementary file 1 [file biomedicines-13-00049-s001.zip › Figure S1.tif]

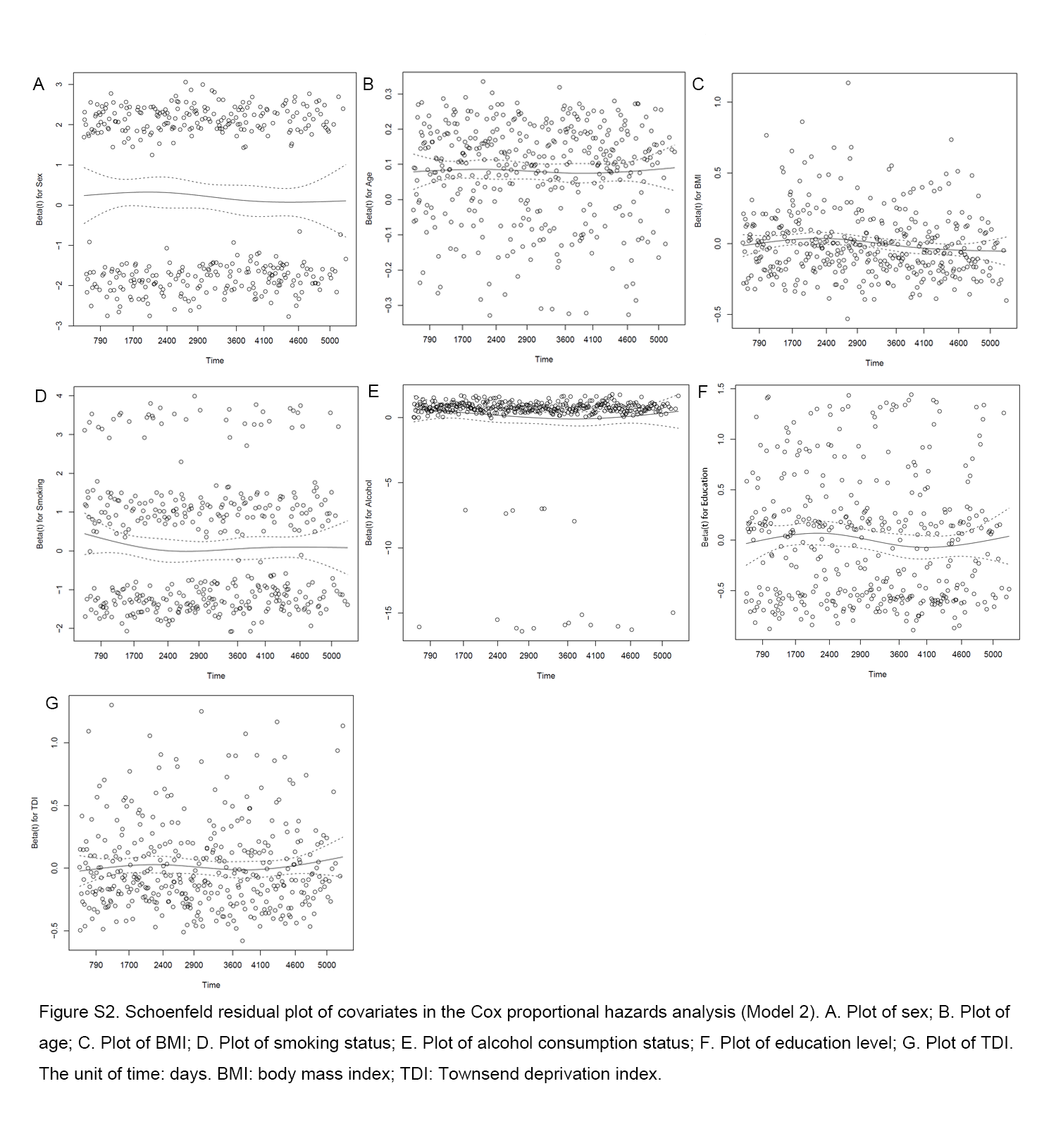

Supplement: Supplementary file 1 [file biomedicines-13-00049-s001.zip › Figure S2.tif]

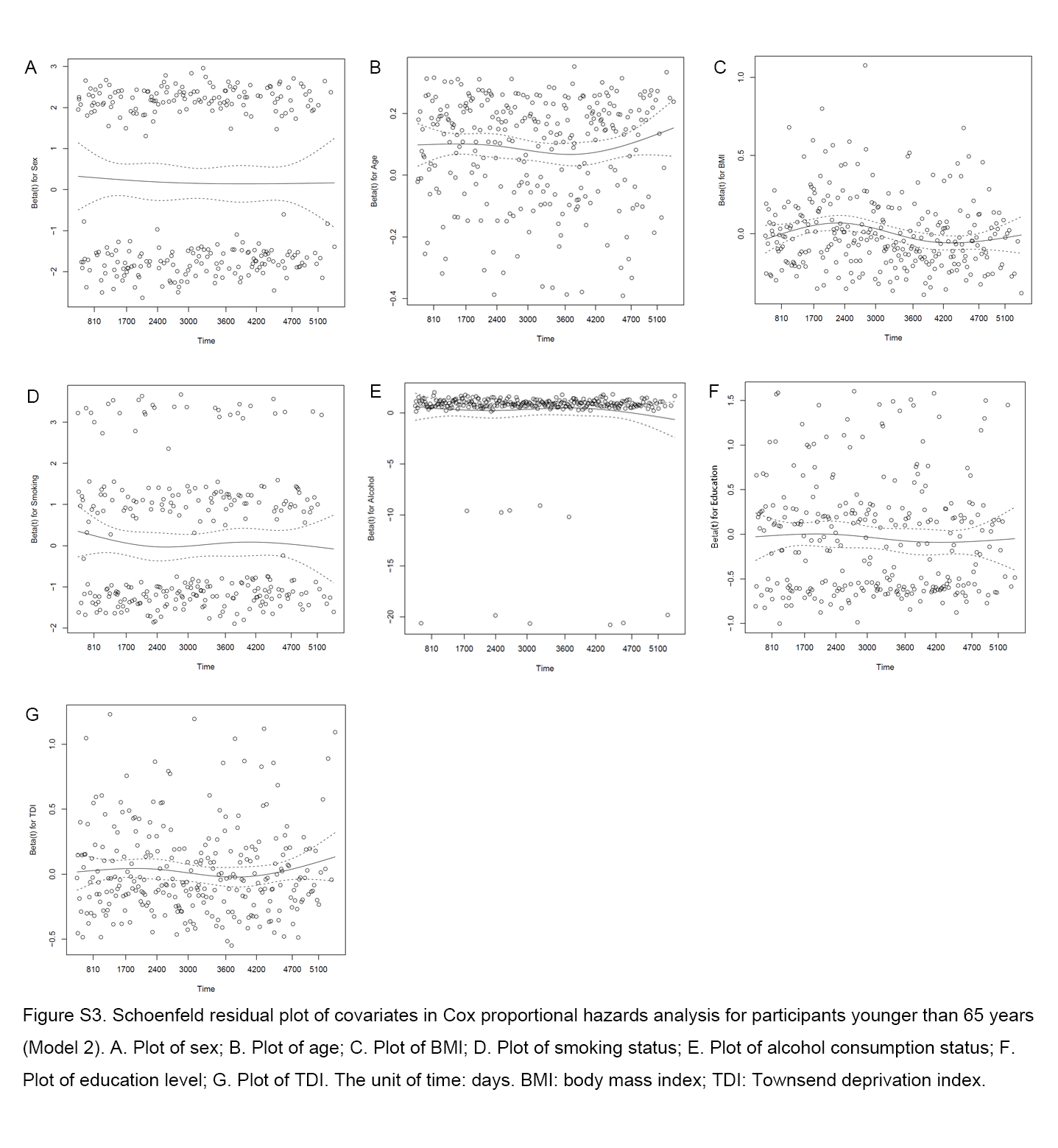

Supplement: Supplementary file 1 [file biomedicines-13-00049-s001.zip › Figure S3.tif]

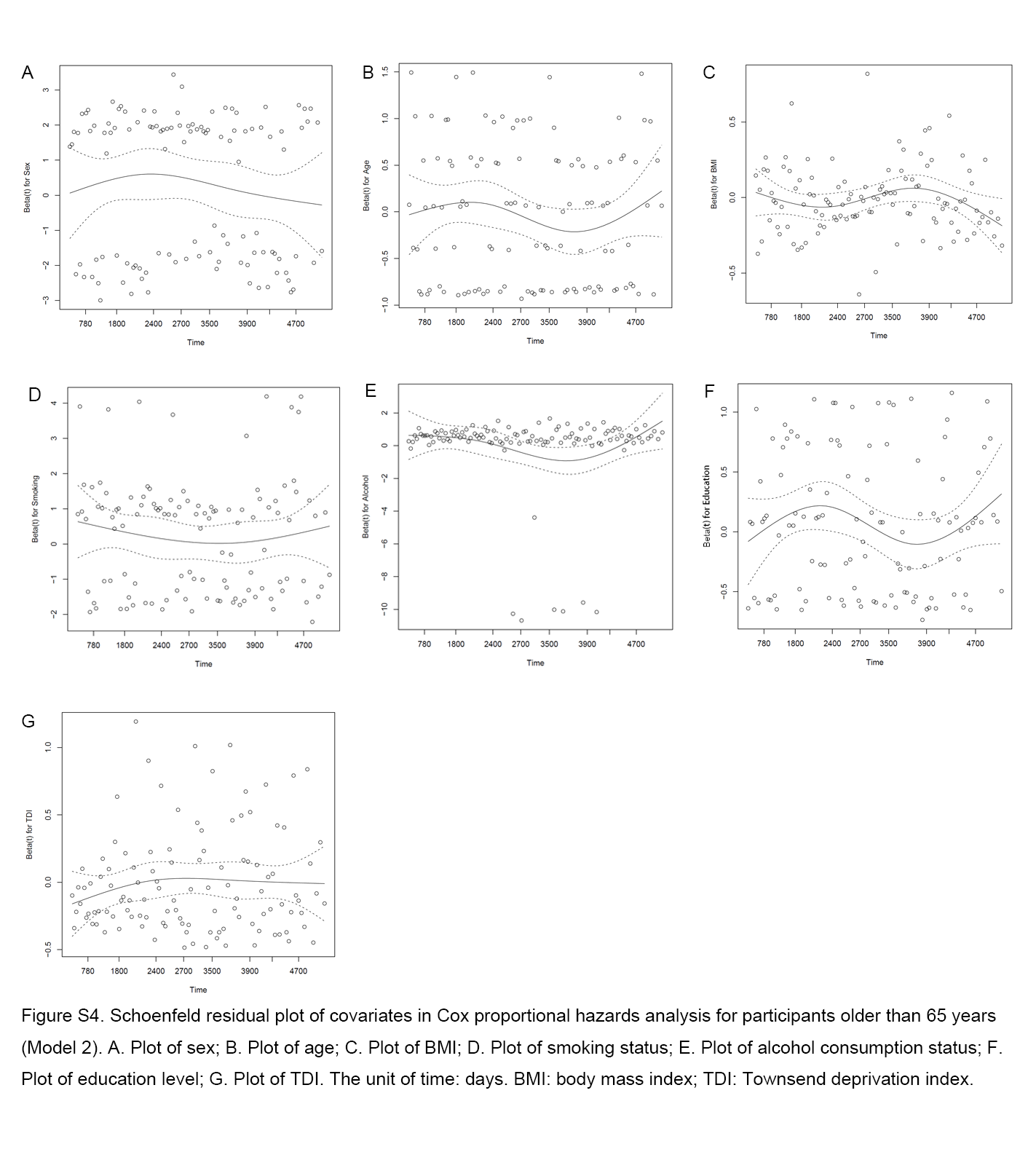

Supplement: Supplementary file 1 [file biomedicines-13-00049-s001.zip › Figure S4.tif]

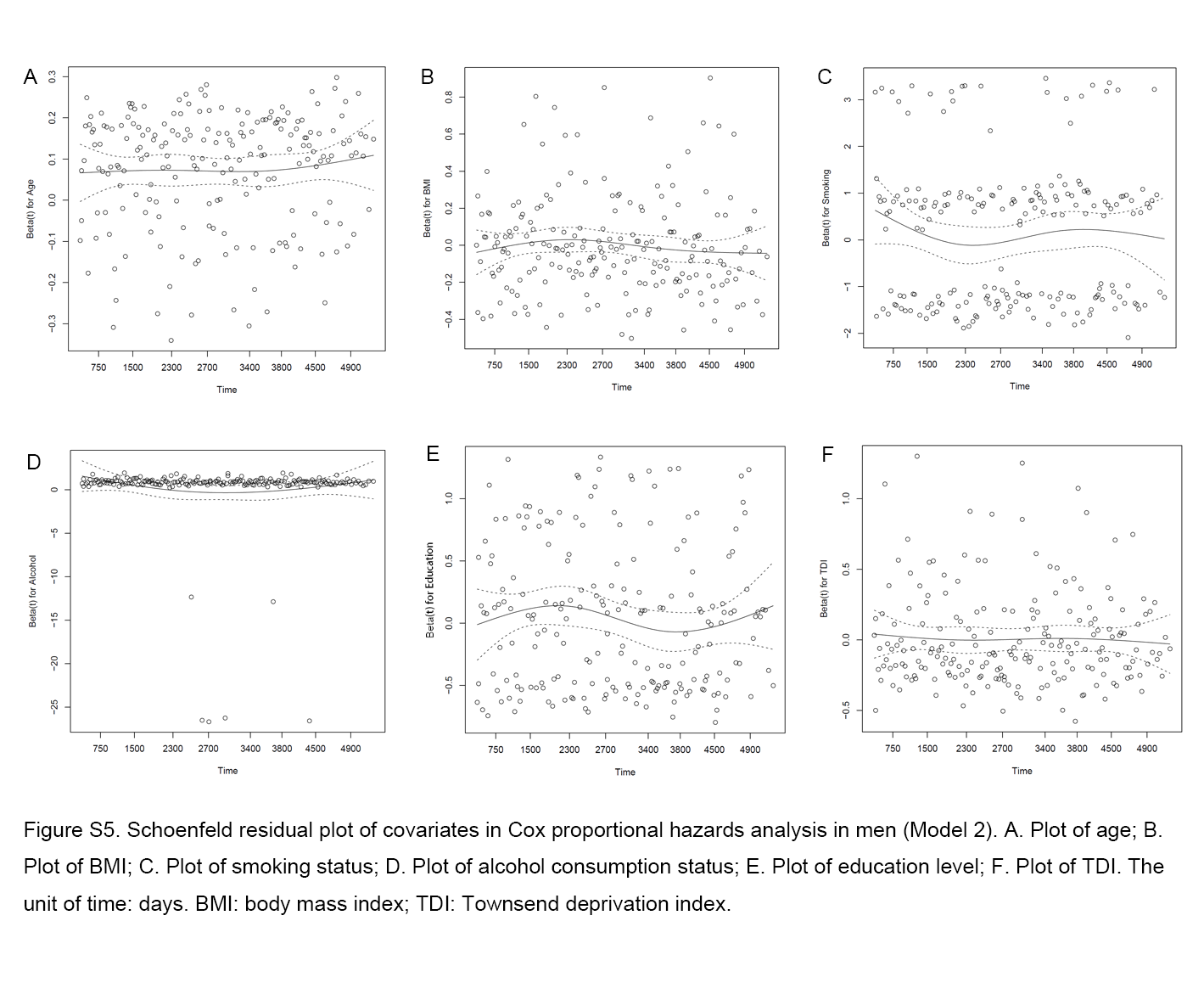

Supplement: Supplementary file 1 [file biomedicines-13-00049-s001.zip › Figure S5.tif]

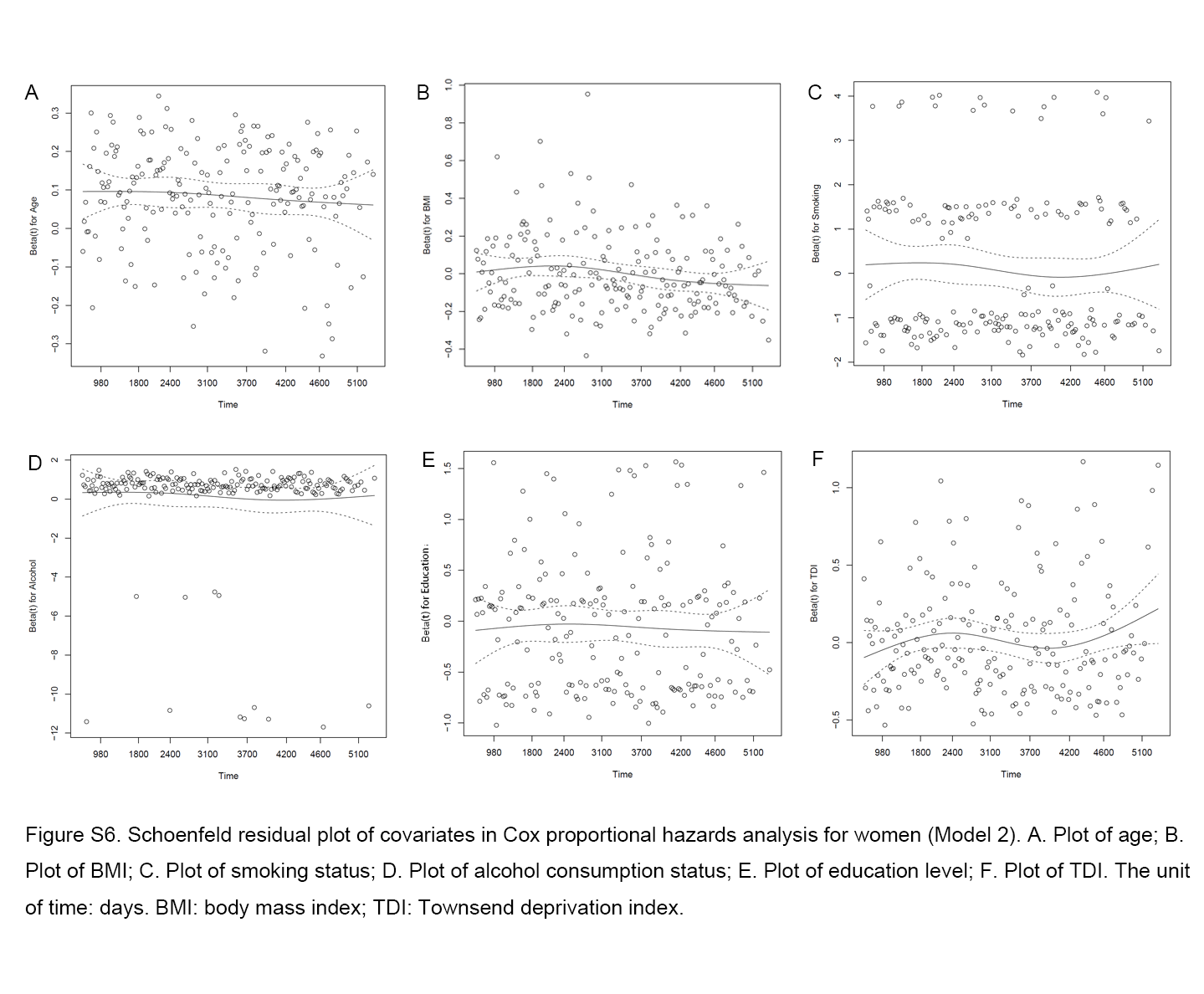

Supplement: Supplementary file 1 [file biomedicines-13-00049-s001.zip › Figure S6.tif]

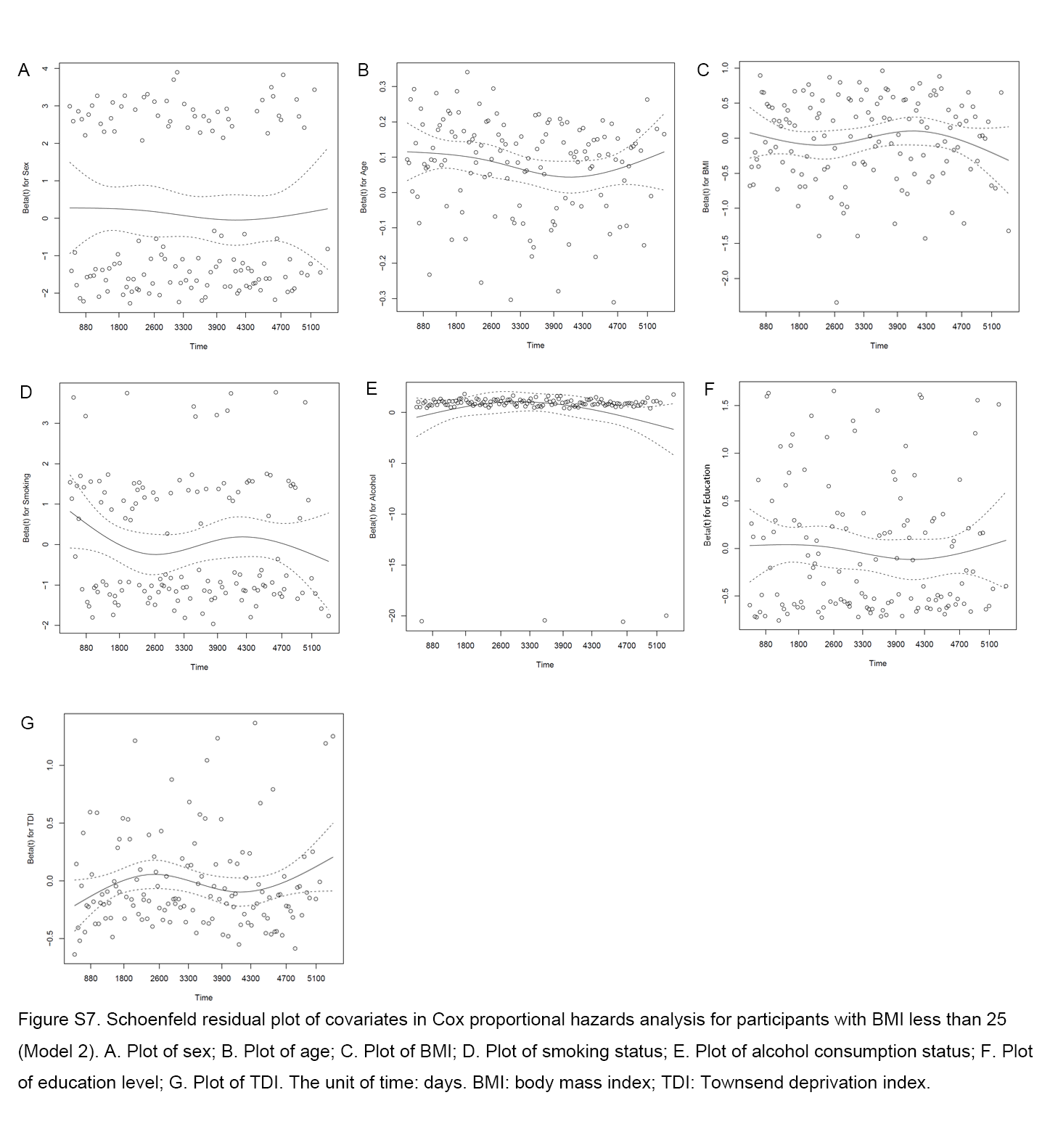

Supplement: Supplementary file 1 [file biomedicines-13-00049-s001.zip › Figure S7.tif]

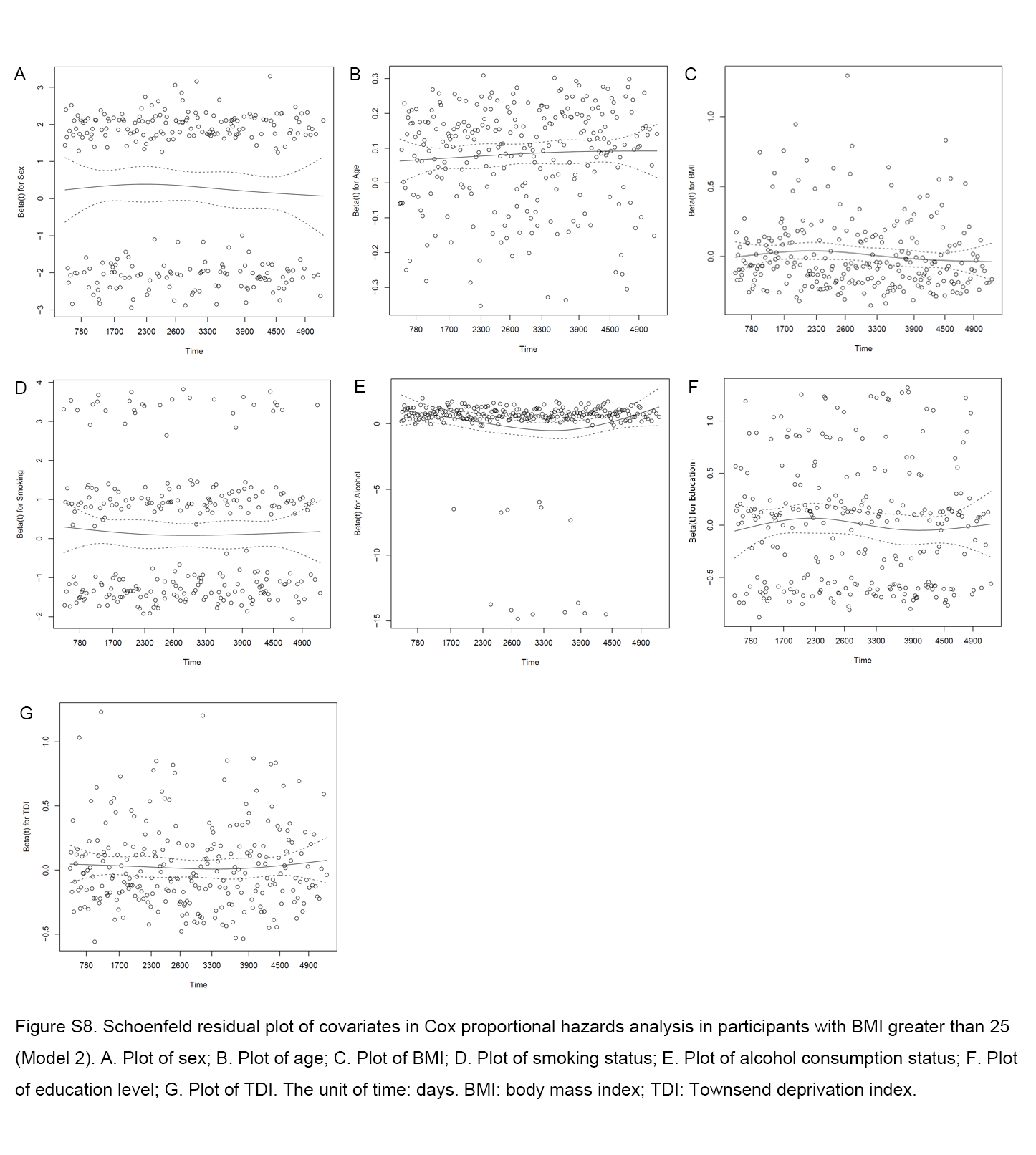

Supplement: Supplementary file 1 [file biomedicines-13-00049-s001.zip › Figure S8.tif]
